# Aviation Security and the TSA's Behavior Detection: Why Effective Academic and Practitioner Dialogue Is Vital

**DOI:** 10.3389/fpsyg.2018.00240

**Published:** 2018-03-06

**Authors:** Vincent Denault, Louise M. Jupe

**Affiliations:** ^1^Département de Communication, Université de Montréal, Montreal, QC, Canada; ^2^Centre D'études en Sciences de la Communication Non Verbale, Montreal, QC, Canada; ^3^Department of Psychology, University of Portsmouth, Portsmouth, United Kingdom

**Keywords:** Transportation Security Administration, security, behavior, nonverbal communication, evidence-based practices, deception detection

On July 20, 2017, under the pen of Kingsbury and Grover, the U.S. Government Accountability Office published a report on the evidence the Transportation Security Administration (hereafter “TSA”) provided to support behavioral indicators used “for identifying passengers who may pose a threat to aviation security” (p. 1). The report's conclusions were unequivocal:

In our review of all 178 sources TSA cited in support of its revised list of behavioral indicators, we found that 98% (175 of 178) of the sources do not provide valid evidence applicable to the specific indicators TSA identified them as supporting (Kingsbury and Grover, 2017, p. 5).

Whilst this report is just one example of how an organization developed and implemented practices that lack scientific evidence, such a report may also suggest an insufficient dialogue, initiated by research scientists, with practitioners to adequately promote scientific knowledge. In this opinion article, our aim is to offer some avenues for thought regarding the reasons why such a dialogue might sometimes appear to be deficient.

Although numerous publications have addressed the subject and offered various definitions (e.g., Gergen et al., 2001; Anderson et al., 2004; Beech et al., 2010; Cooren, 2010), authors generally agree a dialogue is not simply the back and forth communication between two or more people. A dialogue can be defined as an interaction where parties genuinely engage in activities of co-construction “to enable something new to emerge from relaxed and non-judgmental curiosity in order that collective thought becomes coherent” (Beech et al., 2010, p. 1343; see also Bohm, 2003). Therefore, if research scientists and practitioners genuinely want to engage in dialogue “to develop solutions to problems in the world of practice, and thereby generate insights for the world of theory” (Beech et al., 2010, p. 1342), they each must fully appreciate the others knowledge. For research scientists, this includes recognizing the value of the practitioners' experiential knowledge.

Knowledge gained through experience is essential to real-life decision making within uncertain situations, especially when there is little to no science (Shön, 1991; Lowman, 2012; Lilienfeld et al., 2013). However, whilst research scientists may be tempted to question, even undervalue such knowledge, one should keep in mind that experiential and scientific knowledge are different types of knowledge developed through different processes. Experiential and scientific knowledge each have their strengths and weaknesses. For example, rigor can be viewed as a strength for aspects of scientific knowledge yet a weakness within experiential knowledge and relevance often serves as a strength for experiential knowledge but may be lacking within scientific understanding (Bartunek and Rynes, 2014).

Another pivotal issue to the lack of dialogue between research scientists and practitioners may stem from the integration of knowledge. For example, if practitioners lend a disproportionate weight to anecdotal evidence and research scientists overestimate the applicability of their results, there is an immediate limit to what either can learn from the other. To effectively understand such strengths and weaknesses and to make informed decisions, research scientists should adequately relay to practitioners what scientific knowledge is and, what it is not. For example, in justice systems, many practitioners lack the knowledge to distinguish science from pseudoscience (e.g., Redding et al., 2001; Moreno, 2003; Faigman, 2006; Lilienfeld and Landfield, 2008; Tadei et al., 2016). In addition, how can research scientists expect practitioners to embrace scientific knowledge when science often offers uncertainty?

Whilst science is transient and far from perfect, practitioners must understand that scientific knowledge is rigorously examined through the peer review process and subsequently subjected to the scrutiny of a worldwide community of research scientists who can bring invaluable benefit to real-life decision making. On the other hand, “alternative” science, or pseudoscience, rising in popularity and frequently offering grandiose solutions without adequate evidence (Lilienfeld et al., 2003), requires blind trust. Whilst nothing can justify the use of pseudoscience, anecdotal evidence can be informative when there is little to no science regarding a specific topic. Nonetheless, if an issue has been extensively studied by research scientists, the use of anecdotal evidence at the expense of scientific knowledge, more so as a rationale to spend taxpayers' money, should at the very least raise serious questions.

There is no doubt that the TSA as well as other security and law organizations long for better public safety, yet failing to embrace evidence-based practices stemming from research into deception detection can result in dire consequences. Practitioners can, in perfectly good faith, develop and implement procedures and practices that would not stand up to critical examination. Research scientists need to acknowledge that practitioners have a significant belief in their procedures and practices. Therefore, to improve the dialogue with practitioners, research scientists should reduce outright negativity, bring about constructive criticism and take a step forward in understanding the practitioners' procedures and practices as a whole. For example, an organization as crucial as the TSA clearly portrays their justifications for their methods, justifications that can fall outside of the area of expertise of research scientists. Whilst research scientists are often dismissive of their methods, without an improvement in dialogue, a stalemate will be reached in terms of developing security protocols, of which the risks are catastrophic.

Obviously, such a task is not without challenges, starting with the time an improvement in dialogue requires. However, whereas research scientists remain committed to carry out research that will serve in real-life situations, dual narratives to allow both research scientists and practitioners to inform one another is of the utmost importance. Research scientists should therefore engage in a dialogue with the TSA as well as other security and law organizations and invite them to jointly work on improving their current methods and on developing and implementing procedures and practices that will stand up to critical examination.

## Author contributions

All authors listed have made a substantial, direct and intellectual contribution to the work, and approved it for publication.

### Conflict of interest statement

The authors declare that the research was conducted in the absence of any commercial or financial relationships that could be construed as a potential conflict of interest.
